# Integrated metabolomics and transcriptomics analysis reveals the mechanism of Tangbi capsule for diabetic lower extremities arterial disease

**DOI:** 10.3389/fmicb.2024.1423428

**Published:** 2024-07-22

**Authors:** Ye-hao Luo, Da-wei Wang, Jia Yao, Yue Hu, Ling Zhao, Jian-xuan Wen, Jin-ming Zhang, Lu-Lu Wu, Guan-jie Fan, Wei Song

**Affiliations:** ^1^School of Second Clinical Medicine, Guangzhou University of Chinese Medicine, Guangzhou, China; ^2^Department of Endocrinology, The Second Affiliated Hospital of Guangzhou University of Chinese Medicine, Guangzhou, China; ^3^Guangdong Provincial Key Laboratory of Clinical Research on Traditional Chinese Medicine Syndrome, Guangzhou, China; ^4^Shunde Hospital Guangzhou University of Chinese Medicine, Guangzhou, China; ^5^The First Affiliated Hospital of Guangzhou University of Chinese Medicine, Guangzhou, China; ^6^Department of Endocrinology Guangzhou 12th People’s Hospital, Guangzhou, China

**Keywords:** diabetic lower extremities arterial disease, integrated metabolomics, transcriptomics, Tangbi capsule, angiogenesis, vascular endothelial injury

## Abstract

**Objective:**

Tangbi capsule (TBC) is a traditional Chinese medicine prescription, which has the potential to improve the vascular insufficiency of lower extremities and limb numbness in diabetes. However, the potential mechanism remains unknown. This study aims to investigate the pharmacological effects and mechanism of TBC on rats with diabetic lower extremities arterial disease (LEAD).

**Methods:**

The mechanism of TBC on diabetic LEAD was investigated through metabolomics and transcriptomics analysis, and the main components of TBC were determined by mass spectrometry. The efficacy and mechanism of TBC on diabetic LEAD rats were investigated through *in vitro* experiments, histopathology, blood flow monitoring, western blot, and real-time polymerase chain reaction.

**Results:**

Mass spectrometry analysis identified 31 active chemical components in TBC including (2R)-2,3-Dihydroxypropanoic acid, catechin, citric acid, miquelianin, carminic acid, salicylic acid, formononetin, etc. *In vitro* analysis showed that TBC could reduce endothelial cell apoptosis and promote angiogenesis. Histopathological analysis showed that TBC led to an obvious improvement in diabetic LEAD as it improved fibrous tissue proliferation and reduced arterial wall thickening. In addition, TBC could significantly increase the expression levels of HIF-1α, eNOS, and VEGFA proteins and genes while reducing that of calpain-1 and TGF-β, suggesting that TBC can repair vascular injury. Compared with the model group, there were 47 differentially expressed genes in the whole blood of TBC groups, with 25 genes upregulated and 22 downregulated. Eighty-seven altered metabolites were identified from the serum samples. Combining the changes in differentially expressed genes and metabolites, we found that TBC could regulate arginine biosynthesis, phenylalanine metabolism, pyrimidine metabolism, arachidonic acid metabolism, pyrimidine metabolism, arachidonic acid metabolism, nucleotide metabolism, vitamin B6 metabolism and other metabolic pathways related to angiogenesis, immune-inflammatory response, and cell growth to improve diabetic LEAD.

**Conclusion:**

TBC improved vascular endothelial injury, apoptosis, lipid accumulation, liver and kidney function, and restored blood flow in the lower extremities of diabetic LEAD rats. The mechanism of TBC in the treatment of diabetic LEAD may be related to the modulation of inflammatory immunity, lipid metabolism, and amino acid metabolism. This study presented preliminary evidence to guide the use of TBC as a therapy option for diabetic LEAD.

## 1 Introduction

Diabetic lower extremity vascular disease refers to lower extremity arterial disease (LEAD), a main component of peripheral arterial disease. Currently, nearly half a billion people worldwide suffer from diabetes, and the prevalence is expected to increase by more than 50 percent in 25 years. It is estimated that LEAD affects more than 200 million people worldwide (Lazzarini et al., 2018). LEAD is the main cause of foot pain, rupture, gangrene, and amputation in diabetic patients. Early-stage LEAD is often asymptomatic, with only mild limb extremity coldness, numbness, and discomfort. As the lesion progresses, intermittent claudication of the lower extremities may occur. When lower limb ischemia is further aggravated, resting pain and even ischemic ulceration and necrosis of the feet or toes can occur, reducing the quality of life of patients and increasing the risk of myocardial infarction, stroke, and other cardiovascular events (Nativel et al., 2018a).

**Figure 1:**
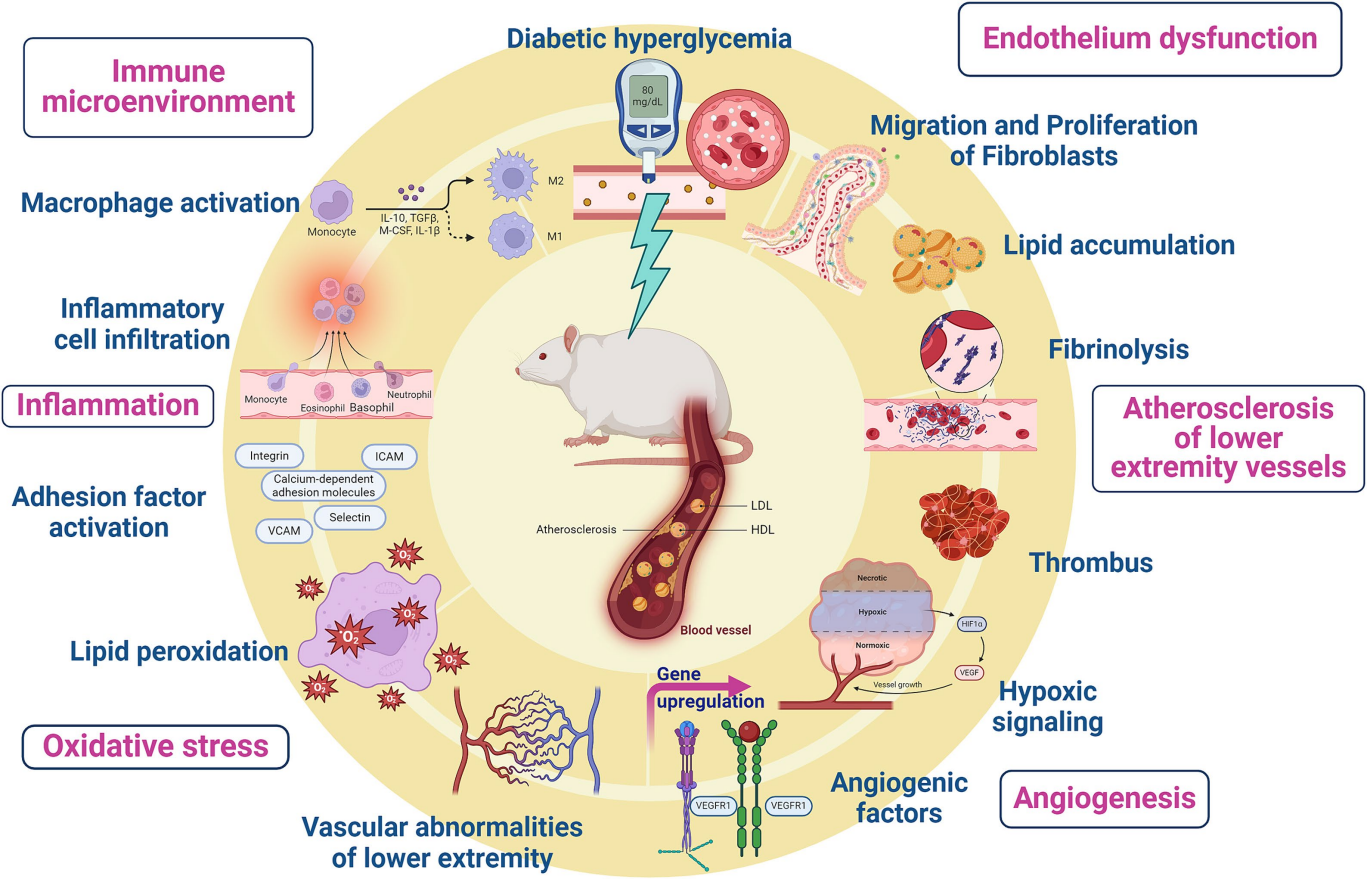
Pathogenesis of diabetic lower extremity vascular disease.

The pathogenesis of diabetic LEAD involves many aspects, including atherosclerotic thrombosis due to metabolic abnormalities, pathological activation of coagulation mechanism, platelet dysfunction and plaque rupture, excessive production of advanced glycosylation end products (AGEs), endothelial dysfunction, oxidative stress, and inflammatory response (Buso et al., 2019) (Figure 1). Current clinical treatment of diabetic LEAD is mainly surgery, anticoagulation, and antiplatelet therapy. Unfortunately, those therapies can only temporarily alleviate the clinical symptoms and fail to restore the function of the lower extremities (LoGerfo and Gibbons, 1996). In addition, most diabetic LEAD patients choose conservative treatment due to the economic burden, and there is no definitive therapeutical regimen that delays the progression of the disease and amputation due to limb gangrene (Weiss and Sumpio, 2006).

Tangbi capsule is a traditional Chinese medicinal preparation for the treatment of diabetic LEAD, which consists of Astragalus membranaceus, Cinnamomum cassiae, Radix Angelicae sinensis, Radix Paeoniae alba, Salviae miltiorrhizae, Radix Rehmanniae praeparata, Lutetiae, Leeches, and Digitoninum. Previous clinical studies have shown that TBC can improve clinical symptoms such as limb coldness, numbness, pain, burning, and skin dryness in diabetic LEAD patients (Hongxia et al., 2007). However, the active ingredients and mechanism of TBC for LEAD are not elucidated. The liquid chromatograph-mass spectrometer (LC-MS) is of great significance for the study of active ingredients in traditional Chinese medicine (Wu et al., 2012). In addition, multi-omics analysis is frequently used to identify potential biomarkers and elucidate disease-related pathogenesis and drug mechanisms of action. Previous studies have shown that the combination of transcriptomics and metabolomics can reveal the distribution and changes of metabolites and genes in serum, contributing to the development of effective therapeutic strategies (Russell et al., 2013).

Therefore, this study aims to clarify the active ingredients of TBC through LC-MS, investigate the therapeutic efficacy and mechanism of TBC on diabetic LEAD through *ex vivo* experiments, and evaluate the role of TBC in regulating the metabolites and the expression of related genes in diabetic LEAD through metabolomics and transcriptomics analysis.

## 2 Materials and methods

### 2.1 Medications and reagents

TBC (Guangdong Pharmaceutical Preparation Approval No. Z20070871, Preparation by Guangdong Provincial Hospital of Traditional Chinese Medicine, Batch No. 06062902) Cilostazol (Chengdu Lier Pharmaceuticals, NMPA Approval No. H20056723), Cilostazol (Chengdu Lier Pharmaceuticals, NMPA Approval No. H20056723), LC/MS grade acetonitrile, methanol, water and formic acid were from Thermo Fisher Scientific (China) Co. Streptomycin and ammonium acetate were from Sigma-Aldrich. Cell Counting Kit-8 (CCK-8) was from Dojindo (Japan). Annexin-V FITC Apoptosis Kit (#556547) was from BD Biosciences Pharmingen (United States). Anti-HIF-1 alpha antibody (ab1), Anti-VEGF Antibody (ab46154), Anti-eNOS antibody (ab300071), Anti-Calpain 1 antibody (ab108400), Anti-iNOS antibody (ab178945), and Anti-TGF beta 1 antibody (ab215715) were from Abcam (United Kingdom).

### 2.2 LC-MS Thermo Q Exactive Orbitrap

0.1 g content powder of TBC was dissolved in 1,000 μL of 80% methanol for a 10-min vortex. The solution was centrifuged at 20,000 × g for 10 min at 4°C. The supernatant was analyzed on a Q Exactive Orbitrap LC-MS system via a ΜLtiMate 3000 RS chromatograph and a Q Exactive high-resolution mass spectrometer.

Mass spectrometry conditions: electrospray ionization source (ESI) detection for FμLl mass/dd-MS2, resolution of 70,000 (fμLl mass), 17,500 (dd-MS2); scan range: 100.0–500.0 m/z; electrospray voltage: 3.2 kV (Positive, Negative); capillary temperature: 300°C; collision gas: high purity argon (purity ≥99.999%); collision energy (N): 30, 40, 60; sheath gas: nitrogen (purity ≥99.999%), 40 Arb; auxiliary gas: nitrogen (purity ≥99.999%), 15 Arb, 350°C; data acquisition time: 30.0 min.

Chromatographic conditions: the column was AQ-C18, 150 × 2.1 mm, 1.8 μm, Welch, at 35°C, flow rate 0.30 mL/min, gradient elution aqueous phase was 0.1% formic acid/water solution (A), the organic phase was methanol (B), the injection volume was 5.00 μL. Chromatographic gradients were (1 min, 2% B; 5 min, 20% B; 10 min, 50% B; 15 min, 80% B; 20–27 min, 95% B; 28–30 min, 2% B). The initial organization of data for high-resolution liquid-quality acquisitions was completed via CD 3.3 (Compound Discoverer 3.3) (Thermo Fisher), and retention time and peak area were used for comparative database (mzCloud) search to determine the main components in TBC (Supplementary Figure S1 and Supplementary Table S1). Detailed sample preparation and instrumental parameter settings can be found in the Supplementary material.

### 2.3 Cell experiments

Human umbilical vein endothelial cells (HUVEC) were obtained from the China Infrastructure of Cell Line Resources and grown under standard conditions (37°C and 5% CO_2_) in DMEM containing 10% (v/v) fetal bovine serum and 100 U/mL penicillin/streptomycin. Subsequent studies were performed at 80% cell fusion. After the successful construction of the cell proliferation model induced by high glucose (25 mmol/L), the cells were divided into normal group, model group, and TBC-containing serum high-, medium- and low-dose groups. The effects of cell survival, angiogenic ability, and apoptosis were investigated.

#### 2.3.1 Cell survival rate

HUVECs were inoculated in 96-well plates, cultured separately according to the experimental grouping. HUVECs were put into the incubator for a 24 h culture, after which 10 μL of CCK-8 solution was added to each well for another 1 h incubation in the incubator. Then, absorbance values at 450 nm were determined by an enzyme counter, and the cell activity was calculated. Cell viability (%) = (OD experimental group − OD blank group)/(OD control group − OD blank group) × 100%.

#### 2.3.2 Angiogenic capacity

HUVEC was intervened with high glucose for 24 h, followed by treatment with TBC-containing serum for 24 h. HUVEC with and without pretreatment (3 × 10^4^ cells/well) were inoculated into matrix gel plates (containing 200 μL of matrix gel; BD Biosciences, San Jose, CA, United States) and incubated at 37°C in 5% CO_2_ for 18 h. Capillary-like structures were evident and counted via an inverted microscope. The vascular network formed by HUVEC was quantified via the Image J plug-in Angiogenesis Analyzer. Data were summarized as mean ± standard deviation (SD).

#### 2.3.3 Apoptosis analysis

Apoptosis was detected by flow cytometry (BD Accuri C6, United States), and HUVECs in 6-well plates were stained with PI and Membrane Associated Protein V-Fluorescein Isothiocyanate according to the manufacturer’s protocols (Zhu et al., 2018). Apoptotic HUVECs were analyzed via FlowJo_V10 software.

### 2.4 Animal experiments and serum sample collection

This animal experiment was ethically approved by the Experimental Animal Ethics Committee of Guangdong Hospital of Chinese Medicine (Approval No. 2023033). Sixty SPF-grade SD male rats, 7 weeks old, weighing 220 ± 10 g, purchased from Guangdong Animal Experiment Center (License No. SYXK(Yue)2018-0094) were used. The experiment was carried out as follows: (1) Twelve rats were evenly divided into blank group, TBC high-dose group 0.316 g/kg, TBC medium-dose group 0.158 g/kg, and TBC low-dose group 0.079 g/kg, and drug-containing serums were prepared to be used for subsequent cellular assays. (2) Eight rats in the control group were fed with conventional feed. Forty rats in the model group were fed with high-fat diet (GD60, mass ratio: 26.2% protein, 26.3% carbohydrate, 34.9% fat; energy supply ratio: 20% protein, 20% carbohydrate, 60% fat). After 8 weeks, the 40 rats were given low-dose STZ (35 mg/kg) intraperitoneally for 2 weeks, after which their right lower limb femoral artery was ligated to establish the model of diabetic LEAD. After the establishment of the model, the rats were randomly divided into model group, positive control group (cilostazol 10 mg/kg^−1^), TBC high-dose group (0.316 g/kg^−1^), TBC medium dose group (0.158 g/kg^−1^) and TBC low-dose group (0.079 g/kg^−1^), with 8 rats in each group. Rats in the control group and model group were given the same amount of purified water once a day for 6 consecutive weeks. At the end of the study, the femoral artery, liver, and kidney tissues of rats in each group were divided into two parts. The first part was rapidly stored at −80°C for WB and qPCR assays, and the second part was fixed with 4% formaldehyde, embedded in paraffin wax, and stained with hematoxylin-eosin (HE) staining, immunofluorescence, and flow cytometry. Serum and whole blood were used for biochemical assays, metabolomics, and transcriptomics analysis.

### 2.5 Serum biochemical analysis

Serum alanine transaminase (ALT), aspartate transaminase (AST), triglyceride (TG), total cholesterol (TC), low-density lipoprotein cholesterol (LDL-C), creatinine (Cr), glucose (GLU), and nitric oxide (NO) levels were measured with commercially available kits (Jiancheng Institute of Bioengineering, Nanjing, China) via a Hitachi 7020 automated biochemical analyzer (Hitachi High-Tech Corporation, Tokyo, Japan). Interleukin-6 (IL-6) (ab234570), tumor necrosis factor-α (TNF-α) (ab236712), IL-1β (ab255730), and endothelin-1 (ET-1) (ab133030) were analyzed with ELISA kits purchased from Abcam (United Kingdom).

### 2.6 HE staining

Freshly collected vascular tissues were fixed in 4% paraformaldehyde (Solarbio, Beijing, China) before embedded in paraffin. Subsequently, the vascular tissue slides were stained with HE according to the manufacturer’s instructions (Solarbio, Beijing, China). A research-grade electric microscope imaging system was used for observation.

### 2.7 Blood perfusion

Lower limb blood flow was detected 28 days after femoral artery ligation modeling. The perfusion unit (PU) of both lower extremities was recorded by a laser scattering microcirculation detection system (PeriCam PSI System) under anesthesia, at constant temperature, and in constant light. Following the anesthesia of 2% pentobarbital sodium intraperitoneal injection, a laser Doppler probe was placed on the soles of the rats, and an area of 3 cm × 5 cm was scanned with the laser diffusion microcirculation imaging. The Doppler effect generated by the movement of blood cells in blood vessels within 1 mm under the skin was converted into current values to draw two-dimensional color perfusion images, where red indicates the maximum perfusion volume, dark blue the minimum, yellow in between, and darker color means smaller perfusion volume. The degree of perfusion in the operated limb was expressed by the blood flow ratio of operated limb/healthy limb for data analysis.

### 2.8 Western blot

Protein samples (25 μg) were separated by 10% SDS-PAGE. Solubilized proteins were transferred to PVDF membranes and blocked in TBST solution containing 5% skimmed milk for 1 h. The cell membranes were incubated with Anti-HIF-1 alpha antibody (1:2,000), Anti-VEGF antibody (1:2,000), Anti-eNOS antibody (1:2,000), Anti-Calpain 1 antibody (1:2,000), Anti-iNOS antibody (1:2,000), and Anti-TGF beta 1 antibody (1:2,000) overnight before being exposed to a secondary antibody at room temperature for 1 h. Cell membranes were visualized via Image Quant LAS-4000 (Genetal Electric, Boston, United States), and relative protein expression levels were determined using Image J.

### 2.9 Quantitative real-time polymerase chain reaction analysis

Total RNA was extracted from femoral artery with Trizol reagent and then reverse transcribed to cDNA with Primescript RT reagent Kit (Takara, Shiga, Japan) according to the manufacturer’s instructions. SYBR Premix Ex Taq (TaKaRa) was applied in a 7500 Real-time PCR System for Real-time PCR analysis (Applied Biosystems, Waltham, United States). Delta–delta Ct was used to calculate the relative mRNA expression. The primer sequences are shown in Table 1.

**Table 1:** The primer sequences.

Gene symbol	Forward primer	Reverse primer
eNOS	TGGCCAAAGTGACCATTGTG	GGCAGGGGACAGGAAATAGT
HIF-1α	TTACAGGATTCCAGCAGACCCA	GCTGATGCCTTAGCAGTGGTC
Calpain-1	CTCATGGATCGAGACGGTAATG	CTTTCGGAAGATGGTCAGGTAA
TGFβ	GGCCAGATCCTGTCCAAGC	GTGGGTTTCCACCATTAGCAC
VEGFA	TAAATCCTGGAGCGTTCACTGTG	TTCGTTTAACTCAAGCTGCCTC
β-actin	ACATCCGTAAAGACCTCTATGCC	TACTCCTGCTTGCTGATCCAC

eNOS, endothelial nitric oxide synthase; HIF-1α, hypoxia inducible factor-1α; TGF-β, transforming growth factor-β; VEGFA, vascular endothelial growth factor.

### 2.10 Non-targeted metabolomic analysis via UHPLC-Q-Exactive MS/MS

The samples (100 μL) were placed in the EP tubes and resuspended with prechilled 80% methanol by well vortex. Samples were then incubated on ice for 5 min and centrifuged at 15,000 g, 4°C for 20 min. Some of the supernatant was diluted to a final concentration containing 53% methanol by LC-MS grade water. The samples were subsequently transferred to a fresh Eppendorf tube and centrifuged at 15,000 g, 4°C for 20 min. Finally, the supernatant was injected into the LC-MS/MS system for analysis. To investigate the stability of the instrument and the reproducibility of the samples, quality control samples were prepared by aliquot pooling. An equal volume of samples was taken from each experimental sample and mixed as quality control samples. Blank samples were prepared with 53% aqueous methanol solution, and the pretreatment process was the same as that of the experimental samples. The mobile phases were A (water +0.1% formic acid +5 mM ammonium acetate) and B (acetonitrile), and a Vanquish LC ultra-high-performance liquid chromatography system (UHPLC) Hypesil Gold column (2.1 × 100 mm, 1.9 μm) (Thermo Fisher, United States) was used to separate the samples.

UHPLC-MS/MS analyses were performed with a Vanquish UHPLC system (Thermo Fisher, Germany) coupled with an Orbitrap Q Exactive^™^ HF-X mass spectrometer (Thermo Fisher, Germany). Samples were injected onto a Hypesil Gold column (100 × 2.1 mm, 1.9 μm) via a 12-min linear gradient at a flow rate of 0.2 mL/min. The eluents for the positive polarity mode were eluent A (0.1% FA in Water) and eluent B (Methanol). The eluents for the negative polarity mode were eluent A (5 mM ammonium acetate, pH 9.0) and eluent B (Methanol). The solvent gradient was set as follows: 2% B, 1.5 min; 2–85% B, 3 min; 85–100% B, 10 min; 100–2% B, 10.1 min; 2% B, 12 min. Q Exactive^™^ HF-X mass spectrometer was operated in positive/negative polarity mode with a spray voltage of 3.5 kV, capillary temperature of 320°C, sheath gas flow rate of 35 psi, and aux gas flow rate of 10 L/min, S-lens RF level of 60, Aux gas heater temperature of 350°C.

Raw LC-MS data were converted to m/z file format using the online platform Wekemo Bioincloud.^1^ Combined with the high-quality mzCloud database constructed from standards paired with mzVault and MassList databases, the molecular signature peaks were matched for identification, which was able to identify as many metabolites as possible in the biological system and maximize the reflection of total metabolite information. Multidimensional statistical analyses (PCA, PLS-DA, and OPLS-DA) were performed using SIMCA-P, and the validity of the OPLS-DA model was verified by the 200-permutation test and evaluated by the *Q*^2^ value. Potential metabolites were screened according to the criteria of variable projection importance (VIP) >1.0 and *p* < 0.05 and evaluated by the HMDB database^2^ and LIPID Maps database^3^ for structural identification. The identified potential metabolites were imported into the online platform Wekemo Bioincloud and based on the KEGG database to reveal the key metabolic pathways.^4^

### 2.11 Transcriptomics

Total RNA was extracted from whole blood of the control group, model group, and TBC-H groups using TRIzol reagent (Invitrogen, CA, United States). The concentration and quality of extracted RNA were measured with an Agilent 2100 bioanalyzer (Agilent Technologies, CA, United States) and Nano Photometer spectrophotometer. The cDNA libraries were constructed and sequenced by Wekemo Tech Guoup Co., Ltd. Shenzhen China using the Illumina NovaSeq platform. The raw data (raw reads) in fastq format were firstly processed through in-house perl scripts. In this step, clean data were obtained by removing low-quality reads and reads containing adapter and ploy-N. At the same time, Q20, Q30, and GC content of the clean data were calculated. All subsequent analyses were based on high-quality clean data. An index of the reference genome was constructed using HISAT2 and paired-end clean reads were also aligned to the rat genome using HISAT2. Transcript abundances were measured as fragments per kilobase of transcript per million fragments mapped (FPKM) using the StringTie. Differentially expressed genes (DEGs) were screened via DESeq2 (version 1.16.1) using the criteria |log2Fold Change| ≥1 and *p*-value <0.05. Gene Ontology (GO) and Kyoto Encyclopedia of Genes and Genomes (KEGG) pathway enrichment analysis of differentially expressed genes was performed with the clusterProfiler R package. PPI analysis of differentially expressed genes was based on the STRING database. A multi-omics correlation heatmap analysis of transcriptome differential genes and differential metabolites was conducted based on the online platform Wekemo Bioincloud (see text footnote 1). The raw RNA sequencing data were deposited in the NCBI database (accession number: PRJNA1088278).

### 2.12 Statistical analysis

All data were expressed as mean ± standard deviation (SD) and statistical analyses were performed on GraphPad Prism 8.3.0 and SPSS 21.0. The Dunnett *t*-test and the Dunnett T3 test were used respectively to test chi-square and heterogeneity of variance. Non-normally distributed data were analyzed using non-parametric tests, and *p* < 0.05 was considered significant.

## 3 Results

### 3.1 Identification of the chemical composition of TBC

LC/MS technology was used to identify the components of TBC. Nine hundred twenty-eight compounds were identified in ESI+ and ESI− modes, with 531 compounds in the mzCloud best match achieving a comprehensive score greater than 60. Thirty-one active compounds were identified based on the Chinese Pharmacopoeia and a review of relevant literature on diabetic LEAD. These chemicals included succinic semialdehyde, D-(+)-Maltose, (2R)-2,3-Dihydroxypropanoic acid, citric acid, catechin, etc. Detailed information on identified compounds is provided in Supplementary Figure S1 and Supplementary Table S1.

### 3.2 Effects of TBC on HUVEC angiogenesis and apoptosis

**Figure 2:**
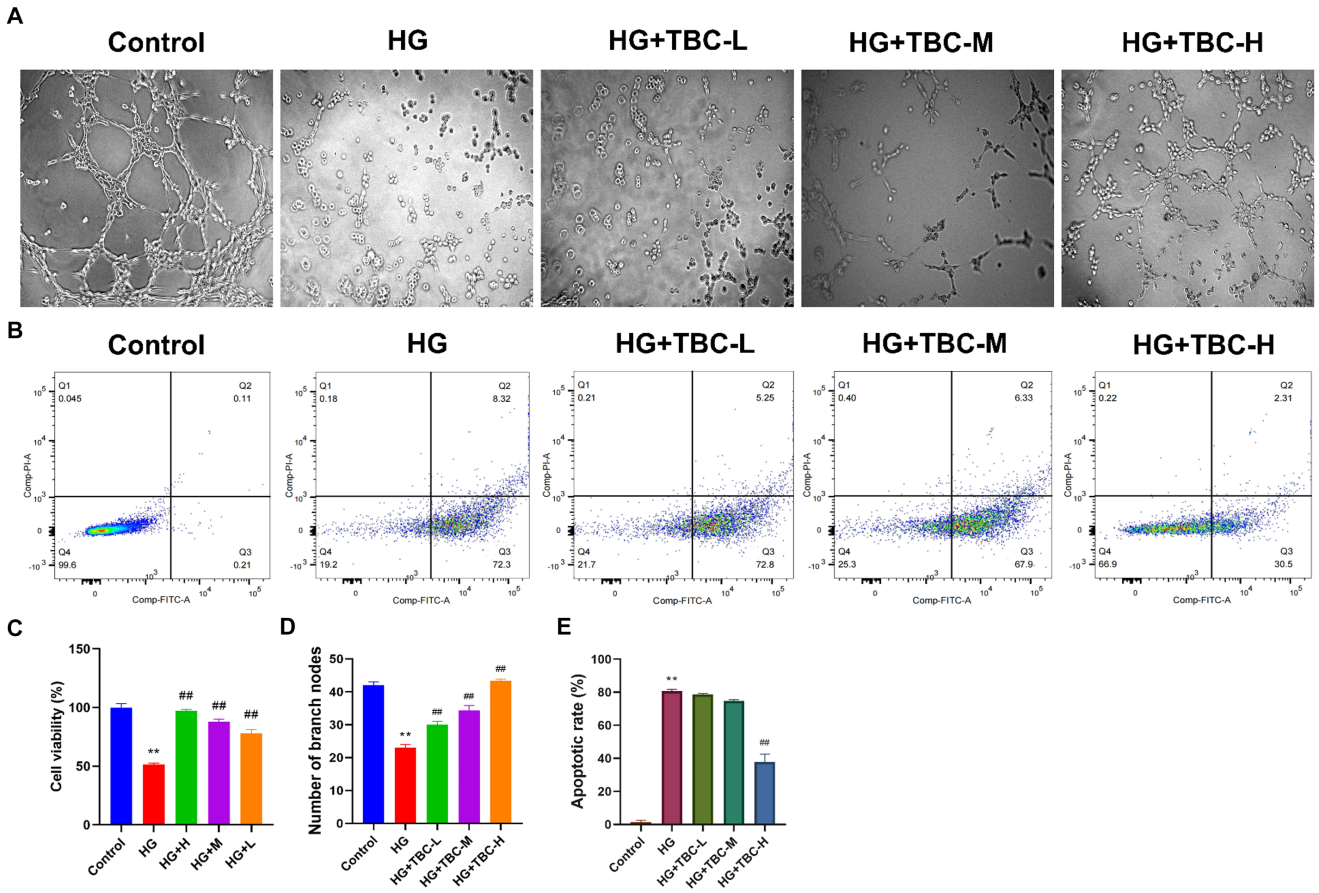
Effect of TBC on HUVEC angiogenesis, cell viability and apoptosis. **(A)** Tube-forming test of sugar paralysis capsule-containing serum. **(B)** Change of apoptosis of sugar paralysis capsule-containing serum by flow cytometry. **(C)** Viability rate of CCK8 cells. **(D)** Bar graph of the tube-forming test. **(E)** Bar graph of apoptosis. Compared with the control group, ^*^p < 0.05 and ^**^p < 0.01 vs. normal group. ^#^*p* < 0.05 and ^##^*p* < 0.01 vs. model group.

To determine whether TBC is involved in HUVEC angiogenesis, high glucose induction was used for modeling endothelial injury caused by lower extremity vascular disease. Results showed that the blank control group had normal vascular structure, while the model group had a significant decrease in the average number of tubular structures. A significant increase was observed in the number of tubular structures in the TBC-containing serum high-, medium-, and low-dose groups (0.316 g/kg, 0.158 g/kg, and 0.079 g/kg) compared with that in the model group, suggesting that TBC may induce vasculature proliferation and promote HUVEC vascularization (Figures 2A–D). In high glucose-treated HUVECs, TBC improved the apoptosis rate, suggesting a protective effect against high glucose-induced apoptosis (Figures 2B,E).

After 24 h of TBC intervention, the Cell Counting Kit-8 (CCK-8) was used to assess the impact on HUVEC viability under high glucose conditions. The results showed that cell viability was significantly reduced to 51.39% in the high glucose-treated model group compared to the control group (*p* < 0.05). However, compared to the high glucose-treated model group, cell viability was elevated by 45.67% (*p* < 0.01), 36.38% (*p* < 0.01), and 26.7% (*p* < 0.01) in HUVECs intervened with high, medium, and low doses of TBC, respectively (Figure 2C).

### 3.3 Effects of TBC on blood flow and biochemical inflammation of diabetic LEAD rats

**Figure 3:**
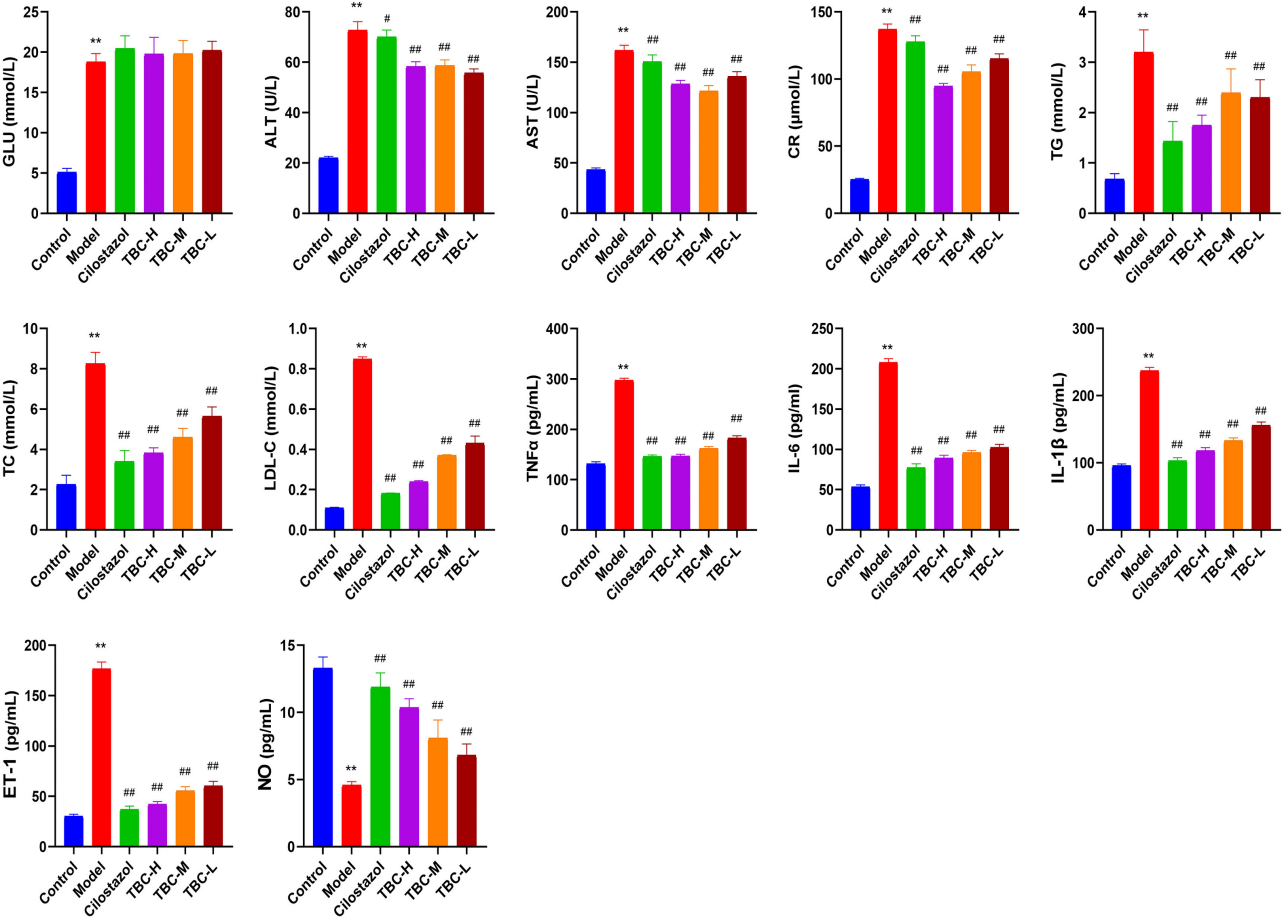
Effect of TBC on biochemical and inflammatory indices in rats with diabetic lower limb vasculopathy. ^*^p < 0.05 and ^**^p < 0.01 vs. normal group. ^#^*p* < 0.05 and ^##^*p* < 0.01 vs. model group.

**Figure 4:**
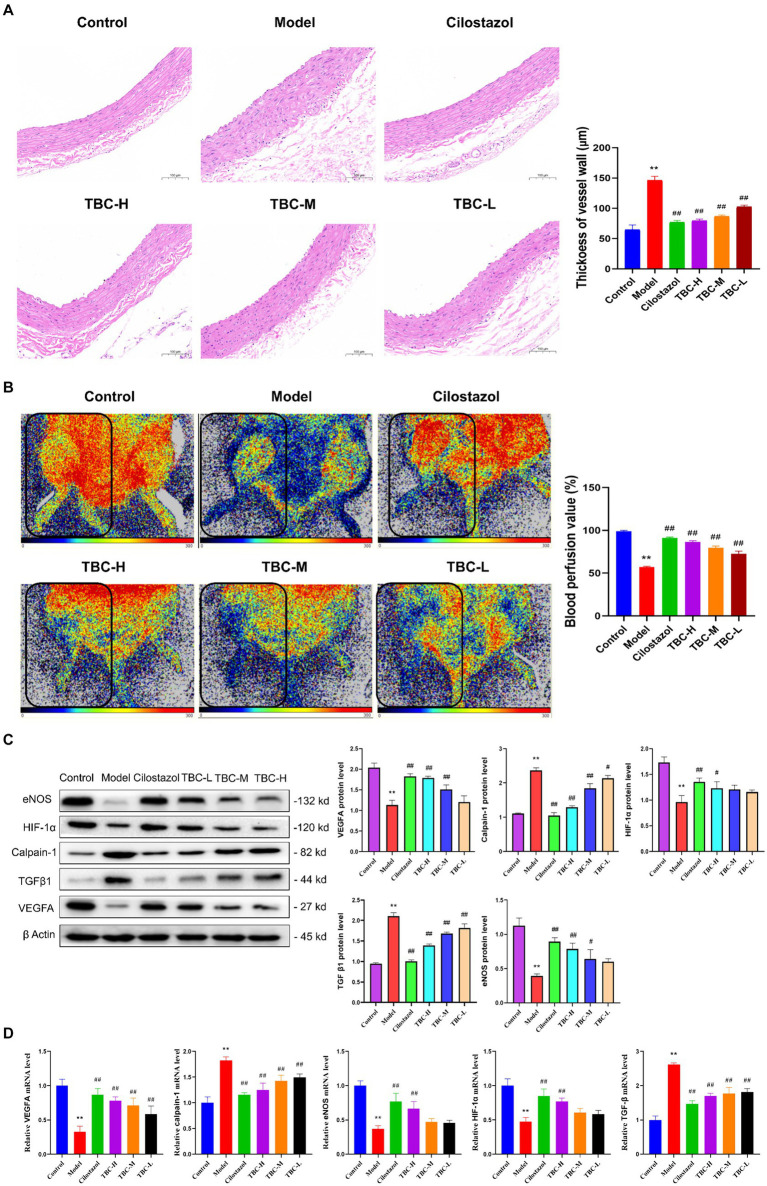
Effect of TBC on histopathology, perfusion volume and related factors in diabetic LEAD rats. **(A)** Pathologic histogram. **(B)** Perfusion after treatment. **(C)** Protein expression. **(D)** Gene expression levels. ^*^p < 0.05 and ^**^p < 0.01 vs. normal group. ^#^*p* < 0.05 and ^##^*p* < 0.01 vs. model group.

Results showed that the serum ALT, AST, TG, TC, LDL-C, CR and GLU levels in the model group were significantly higher than those in the blank group, while NO levels were significantly lower than those in the blank group. After 6 weeks of intervention, the levels of ALT, AST, TG, TC, LDL-C, and CR were significantly reduced, as were the inflammatory factors IL-6, TNFα, and IL-1β. However, NO levels were significantly elevated. There was no significant change in serum GLU levels (Figure 3). In addition, HE staining of femoral artery tissue sections showed that the femoral artery wall was not thickened in the normal group, with no plaque formation or detachment. In the model group, there was plaque accumulation in the arterial wall, marked thickening of the vessel wall, disorganized cell arrangement, and marked intimal hyperplasia. There was slight arterial wall thickening in the low-dose group of TBC, and the cell arrangement was poor. There were no necrotic cells in the middle-dose group of TBC, and the proliferative fibrous tissues were improved. There were no necrotic cells in the high-dose group of TBC and the group of cilostazol, and the cells were neatly arranged and structurally intact (Figure 4A). In addition, diabetic lower extremity blood flow was significantly increased in the cilostazol, TBC-H, TBC-M, and TBC-L groups compared with the model group (Figure 4B).

To further explore the effects of TBC on diabetic LEAD rats, Western blot and PCR were used to detect the levels of related proteins and gene expression in the lower extremity femoral artery tissue. The results showed that after 6 weeks, the expression levels of HIF-1α, eNOS, VEGFA proteins and genes were significantly increased in the TBC-H, TBC-M, TBC-L, and positive control groups compared with the model group, but calpain-1 and TGFβ were significantly decreased (Figures 4C,D).

These results suggest that TBC improves vascular injury and promotes blood flow recovery in diabetic LEAD rats.

### 3.4 Analysis of TBC on serum metabolomics in diabetic LEAD rats

**Figure 5:**
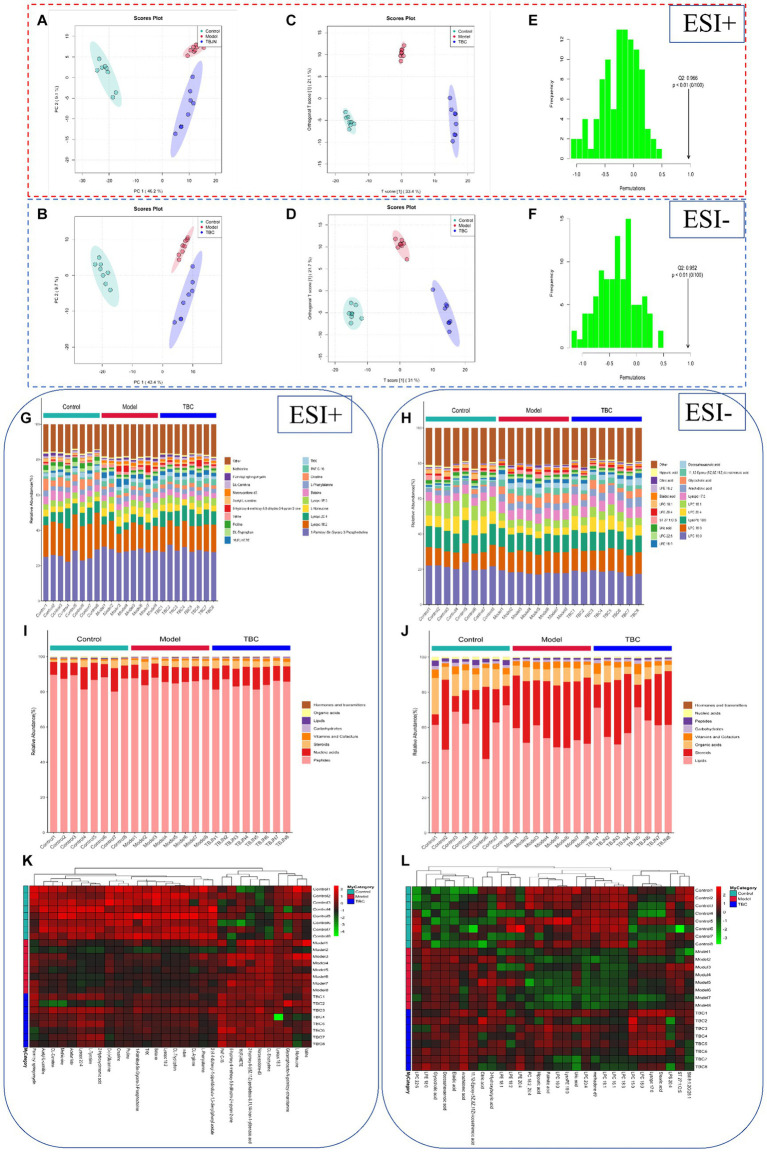
Effect of TBC on serum metabolomic profiles in diabetic LEAD rats. **(A,B)** Establishment of the principal component analysis score plot based on the metabolites of serum in the control, model, and TBC groups (student’s *t*-test, *p* < 0.05, FC >1.0). **(A)** Positive ion. **(B)** Negative ion. **(C,D)** *In situ* partial least squares discriminant analysis scoring plots. (C) Positive ion. (D) Negative ion. **(E,F)** Alignment test for OPLS-DA scoring plots. **(E)** Positive ion. **(F)** Positive ion. Positive-negative ion mode: **(G,H)** top 20 metabolite percentage stacked histograms. **(I,J)** Metabolite percentage stacked histograms that play biological roles. **(K,L)** Metabolite heat map clustering results.

Both animal and cellular results showed that the TBC high-dose group was more effective than the medium and low-dose groups in a concentration-dependent manner. To further explore the mechanism of TBC in the treatment of diabetic lower extremity arterial disease, the blank group, model group, and TBC-H (8 samples in each group) group were selected for metabolomic analysis of serum through an untargeted metabolomics approach. PCA modeling was used to obtain data overview, detect outliers, and evaluate metabolomic differences. The PCA score plot in positive and negative ion mode showed a clear trend of separation in the blank group and the model group, indicating that the metabolites were significantly disturbed. 6-week TBC treatment was significantly separated from the model group, suggesting that TBC altered the metabolic status of diabetic lower extremity arterial disease (Figures 5A,B). An OPLS-DA model was constructed to investigate differential metabolites. One thousand substitution tests yielded intercept values of *R*^2^ = 0.998 and *Q*^2^ = −0.966 in the positive ion mode and *R*^2^ = 0.988 and *Q*^2^ = 0.952 in the negative ion mode, indicating that the model had significant discriminative and predictive capability. (Figures 5C–F). Comparison of metabolite composition structures between subgroups showed that there were mainly 1-Palmitoyl-Sn-Glycero-3-Phosphocholine, Lysopc (18:2, 20:4, 18:3), L-Norleucine, Betaine, L-Phenylalanine dominant compounds (Figure 5G) in the positive ion mode, and Lysopc (16:0, 18:0, 20:4, 18:1, 17:0), LysoPE 18:0 and Arachidonic acid dominant compounds in the negative ion mode (Figure 5H). The biological functions of these metabolites were mainly related to peptides, nucleic acids, steroids, vitamins and cofactors, organic acids, and lipids (Figures 5I,J). To observe the distribution of metabolites in each sample, a heat map of top 30 relative metabolites was generated, and it was found that the samples in different subgroups clustered to different positions, indicating that significant differences in the metabolite compositions of the three groups, and significant structural alterations in some of the metabolite compositions (Figures 5K,L).

**Figure 6:**
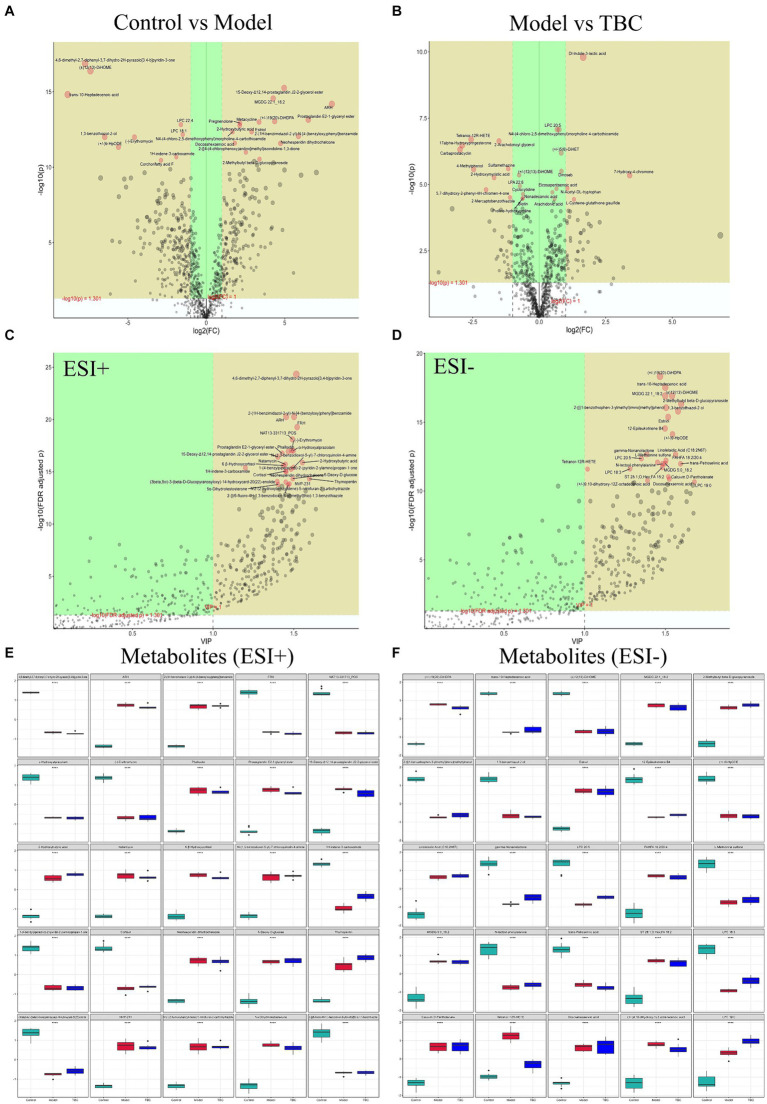
Screening of TBC for differential metabolites in diabetic LEAD rats. Positive and negative ion mode: **(A)** Volcano plot of differential metabolite changes in model and control groups. **(B)** Volcano plots of differential metabolite changes in TBC and model groups. **(C,D)** PLS-DA metabolite significance plots. **(C)** Positive ion. **(D)** Negative ion. **(E,F)** Metabolite difference box plots. **(E)** Positive ion. **(F)** Negative ion.

**Figure 7:**
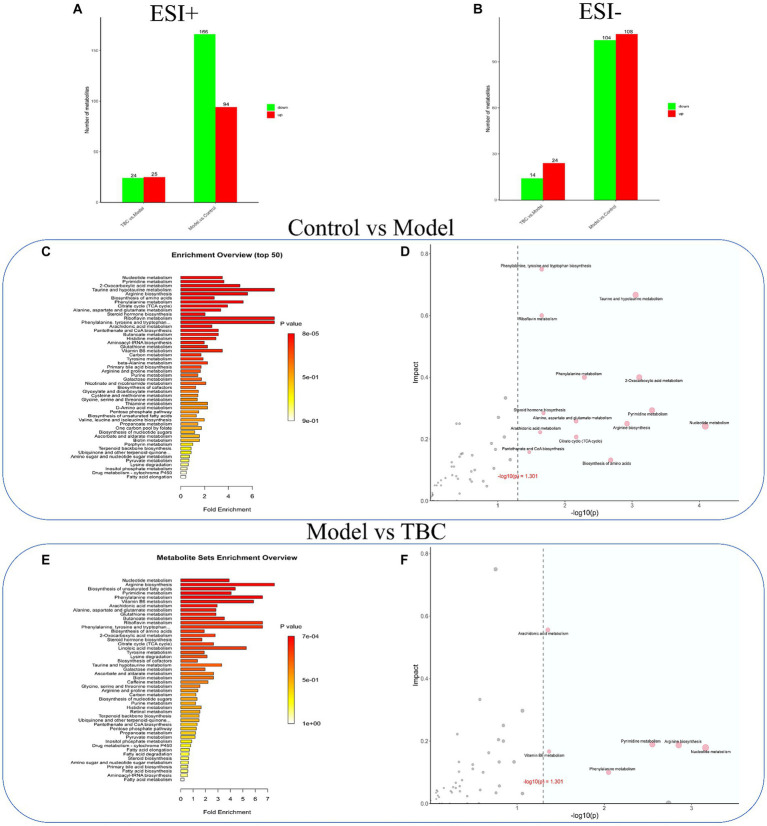
Effect of TBC on serum metabolic pathways in diabetic LEAD rats. Positive and negative ion modes: **(A,B)** Number and trend of metabolites regulated by TBC. **(A)** Positive ion. **(B)** Negative ion. **(C,D)** Metabolic pathway analyses of KEGG-enriched differential serum metabolites in model and control groups (*p* < 0.05). **(E,F)** Metabolic pathway analysis of KEGG-enriched differential serum metabolites in TBC and model groups (*p* < 0.05).

Volcano plots, and metabolite importance plots were constructed to better capture the metabolic changes. Volcano plots showed the trends of differential metabolite expression between the control group and the model group and between the model group and TBC group (Figures 6A,B). The metabolite importance plots showed the trends of differential metabolite expression in the positive and negative modes (Figures 6C,D), with compounds meeting the conditions of VIP >1, *p* < 0.05, FC ≥1 condition considered differential metabolites. To visualize the differences in metabolites between groups, box plots of the top-ranked representative differential metabolites were constructed (Figures 6E,F). In both negative and positive ion modes, a total of 472 dysregulated metabolites were identified in the model group compared with the control group, with 202 metabolites up-regulated and 270 down-regulated compared. Eighty-seven dysregulated metabolites were identified in the TBC group compared with the model group, with 49 metabolites up-regulated and 38 metabolites down-regulated (Figures 7A,B). Based on the metabolites shared in the Con, model, and TBC groups, TBC intervention in diabetic LEAD mainly regulated LPC 20:3, Dl-Indole-3-lactic acid, Deoxycholic acid, Tetranor-12R-HETE, Prostaglandin D2, Ergosta-5,7,9(11),22-Tetraen-3-beta-Ol, Ouabain glutathione disulfide, LNH, Cytidine 5′-Monophosphate-N-Acetylneuraminic Acid and 59 other differential metabolites (Table 2). Significant differential metabolites were selected and overrepresentation analysis (ORA) was used to identify KEGG pathways significantly enriched in these metabolites and to measure topological effects. The results showed that differential metabolites between control and model groups were associated with pathways such as phenylalanine, tyrosine, and tryptophan biosynthesis, taurine and hypotaurine metabolism, riboflavin metabolism, phenylalanine metabolism, nucleotide metabolism, and arginine biosynthesis (Figures 7C,D). After TBC intervention, TBC restored arginine biosynthesis, phenylalanine metabolism, pyrimidine metabolism, arachidonic acid metabolism, and nucleotide metabolism. Vitamin B6 metabolism pathway was also regulated (Figures 7E,F).

**Table 2:** Differential metabolites in serum samples in positive and negative ion modes.

No.	Metabolite	ESI mode	Formula	Molecular weight	RT (min)	m/z	Normal/Model	Model/TBC
1	LPC 20:3	−	C_28_H_52_NO_7_P	591.35477	9.412	590.3475	↓^**^	↑^##^
2	Dl-Indole-3-lactic acid	−	C_11_H_11_NO_3_	205.0736	5.63	204.0663	↓^**^	↑^##^
3	Deoxycholic acid	−	C_24_H_40_O_4_	392.29321	7.027	391.2859	↓^**^	↑^#^
4	Tetranor-12R-HETE	−	C_16_H_26_O_3_	266.18866	7.459	265.1814	↑^**^	↓^##^
5	Prostaglandin D2	−	C_20_H_32_O_5_	334.21513	7.241	333.2079	↑^**^	↓^##^
6	2-Hydroxymyristic acid	−	C_14_H_28_O_3_	244.20393	8.109	243.1967	↑^**^	↓^##^
7	2′-Deoxycytidine	−	C_9_H_13_N_3_O_4_	227.09082	1.384	262.0603	↓^**^	↑^##^
8	12-Hydroxydodecanoic acid	−	C_12_H_24_O_3_	216.1725	7.251	215.1652	↑^**^	↓^##^
9	Lithocholic acid	−	C_24_H_40_O_3_	376.29816	8.751	375.2908	↓^**^	↑^##^
10	Corchorifatty acid F	−	C_18_H_32_O_5_	328.22562	6.668	327.2183	↓^**^	↑^#^
11	LNAPE 20:5/N-18:0	−	C_43_H_76_NO_8_P	765.52638	11.134	764.5191	↓^**^	↑^##^
12	L-Glutathione (reduced)	−	C_10_H_17_N_3_O_6_S	307.08409	1.907	306.0768	↓^**^	↑^##^
13	AICA ribonucleotide	−	C_9_H_15_N_4_O_8_P	338.06174	3.826	383.06	↓^**^	↑^##^
14	Estrone sulfate	−	C_18_H_22_O_5_S	350.12152	5.371	349.1142	↓^*^	↑^##^
15	2,3-Dinor-TXB2	−	C_18_H_30_O_6_	342.20462	6.379	341.1973	↑^**^	↓^#^
16	LPC 20:0	−	C_28_H_58_NO_7_P	597.4019	11.012	596.3946	↓^**^	↑^##^
17	N-Acetyl-Asp-Glu	−	C_13_H_14_N_2_O_3_	246.10065	5.543	245.0934	↑^*^	↓^##^
18	Hippuric acid	−	C_9_H_9_NO_3_	179.05766	5.336	178.0504	↓^*^	↑^##^
19	3-Hydroxydecanoic acid	−	C_10_H_20_O_3_	188.14078	6.545	187.1335	↑^**^	↓^##^
20	2,5-Dimethylphenol	−	C_8_H_10_O	122.07227	5.546	121.065	↑^**^	↓^##^
21	5-Sulfosalicylic acid	−	C_7_H_6_O_6_S	217.98839	4.854	216.9811	↑^**^	↓^##^
22	Prostaglandin H2	−	C_20_H_32_O_5_	334.2124	7.979	333.2051	↓^**^	↑^#^
23	L-Cysteine-glutathione gisulfide	−	C_13_H_22_N_4_O_8_S_2_	426.08837	1.365	425.0811	↓^**^	↑^##^
24	N-Acetyl-DL-tryptophan	−	C_11_H_16_N_2_O_8_	304.09067	6.438	303.0834	↓^**^	↑^##^
25	(±)9-HpODE	−	C_18_H_32_O_4_	312.23051	7.98	311.2232	↓^**^	↑^#^
26	Glutathione	−	C_10_H_17_N_3_O_6_S	307.08395	1.375	306.0767	↓^**^	↑^##^
27	LPE O-20:2	−	C_25_H_50_NO_6_P	491.33841	10.621	490.3311	↓^**^	↑^##^
28	Indole-2-carboxylic acid	−	C_9_H_7_NO_2_	161.04708	5.27	160.0397	↓^*^	↑^##^
29	10-Hydroxydecanoic acid	−	C_10_H_20_O_3_	188.14079	5.995	187.1335	↓^**^	↑^##^
30	7-Hydroxy-4-chromone	−	C_9_H_6_O_3_	162.03096	5.828	161.0242	↓^**^	↑^##^
31	Ergosta-5,7,9(11),22-Tetraen-3-beta-Ol	+	C_15_H_10_O_4_	254.05813	5.743	255.0654	↑^**^	↓^#^
32	Ouabain	+	C_29_H_44_O_12_	566.27392	8.551	567.2812	↑^*^	↓^#^
33	Glutathione disulfide	+	C_20_H_32_N_6_O_12_S_2_	612.15061	2.073	307.0826	↓^**^	↑^##^
34	LNH	+	C_16_H_26_N_6_O_5_	382.19646	4.726	383.2037	↓^**^	↑^#^
35	Cytidine 5′-monophosphate-N-acetylneuraminic acid	+	C_20_H_31_N_4_O_16_P	614.14616	2.296	308.0804	↓^**^	↑^##^
36	4-(3,4-dihydro-2H-1,5-benzodioxepin-7-yl)-2-methyl-1,3-thiazole	+	C_13_H_13_NO_2_S	225.08293	6.005	248.0721	↓^**^	↓^##^
37	Histamine	+	C_5_H_9_N_3_	111.08008	1.144	112.0874	↓^**^	↑^##^
38	17alpha-Hydroxyprogesterone	+	C_21_H_30_O_3_	330.21934	5.945	331.2266	↑^**^	↓^##^
39	L-Glutathione oxidized	+	C_20_H_32_N_6_O_12_S_2_	612.15077	1.401	307.0826	↓^**^	↑^##^
40	Epigallocatechin	+	C_15_H_14_O_7_	306.07524	1.401	307.0825	↓^**^	↑^##^
41	Stearoyl ethanolamide	+	C_20_H_41_NO_2_	310.28733	10.721	311.2946	↓^**^	↓^#^
42	Carbaprostacyclin	+	C_21_H_34_O_4_	332.23534	5.999	333.2426	↑^**^	↓^##^
43	4-hydroxy-3-(3-methylbut-2-en-1-yl)benzoic acid	+	C_12_H_14_O_3_	206.09224	6.17	207.0995	↓^**^	↑^##^
44	4-(4-methoxyphenyl)-2-(2-thienyl)pyrimidine	+	C_15_H_12_N_2_OS	268.06635	1.398	269.0736	↑^**^	↓^##^
45	Guanosine monophosphate	+	C_10_H_14_N_5_O_8_P	363.05739	2.744	364.0647	↑^**^	↓^#^
46	Levodopa	+	C_9_H_11_NO_4_	197.06884	4.987	180.0656	↓^**^	↑^##^
47	7-Hydroxy-3,4-dihydrocarbostyril	+	C_9_H_9_NO_2_	163.06343	5.32	164.0707	↓^**^	↑^##^
48	Lysopc 20:0	+	C_28_H_58_NO_7_P	551.39506	10.742	552.4021	↓^**^	↑^##^
49	YQH	+	C_20_H_26_N_6_O_6_	892.37627	7.332	893.3836	↑^**^	↓^#^
50	2-Arachidonoyl glycerol	+	C_23_H_38_O_4_	395.30321	7.102	396.3105	↑^**^	↓^##^
51	2-Mercaptobenzothiazole	+	C_7_H_5_NS_2_	166.98655	6.123	167.9938	↓^**^	↓^##^
52	PC(16:0/16:0)	+	C_40_H_80_NO_8_P	733.5598	9.525	734.5671	↓^*^	↓^#^
53	Naringin dihydrochalcone	+	C_27_H_34_O_14_	582.19238	5.247	583.1997	↑^**^	↑^#^
54	5,7-dihydroxy-2-phenyl-4H-chromen-4-one	+	C_15_H_10_O_4_	254.05813	5.743	255.0654	↑^**^	↓^##^
55	Lysopc 20:4	+	C_28_H_50_NO_7_P	543.33197	8.853	544.3392	↓^**^	↑^##^
56	3-Methoxy prostaglandin F1α	+	C_21_H_38_O_6_	408.24886	7.533	409.2563	↓^**^	↑^##^
57	Hydrocortisone	+	C_21_H_30_O_5_	362.20919	5.987	385.1986	↓^*^	↑^##^
58	Levothyroxine	+	C_15_H_11_I_4_NO_4_	776.68632	6.152	777.6936	↓^**^	↑^##^
59	Celastrol	+	C_29_H_38_O_4_	468.28738	6.246	451.2839	↑^**^	↓^##^

↓, down-regulation; ↑, up-regulation; /, no significance. ^*^p < 0.05 and ^**^p < 0.01 vs. normal group. ^#^*p* < 0.05, ^##^*p* < 0.01 vs. model group.

### 3.5 Analysis of TBC on whole blood transcriptomics in diabetic LEAD rats

**Figure 8:**
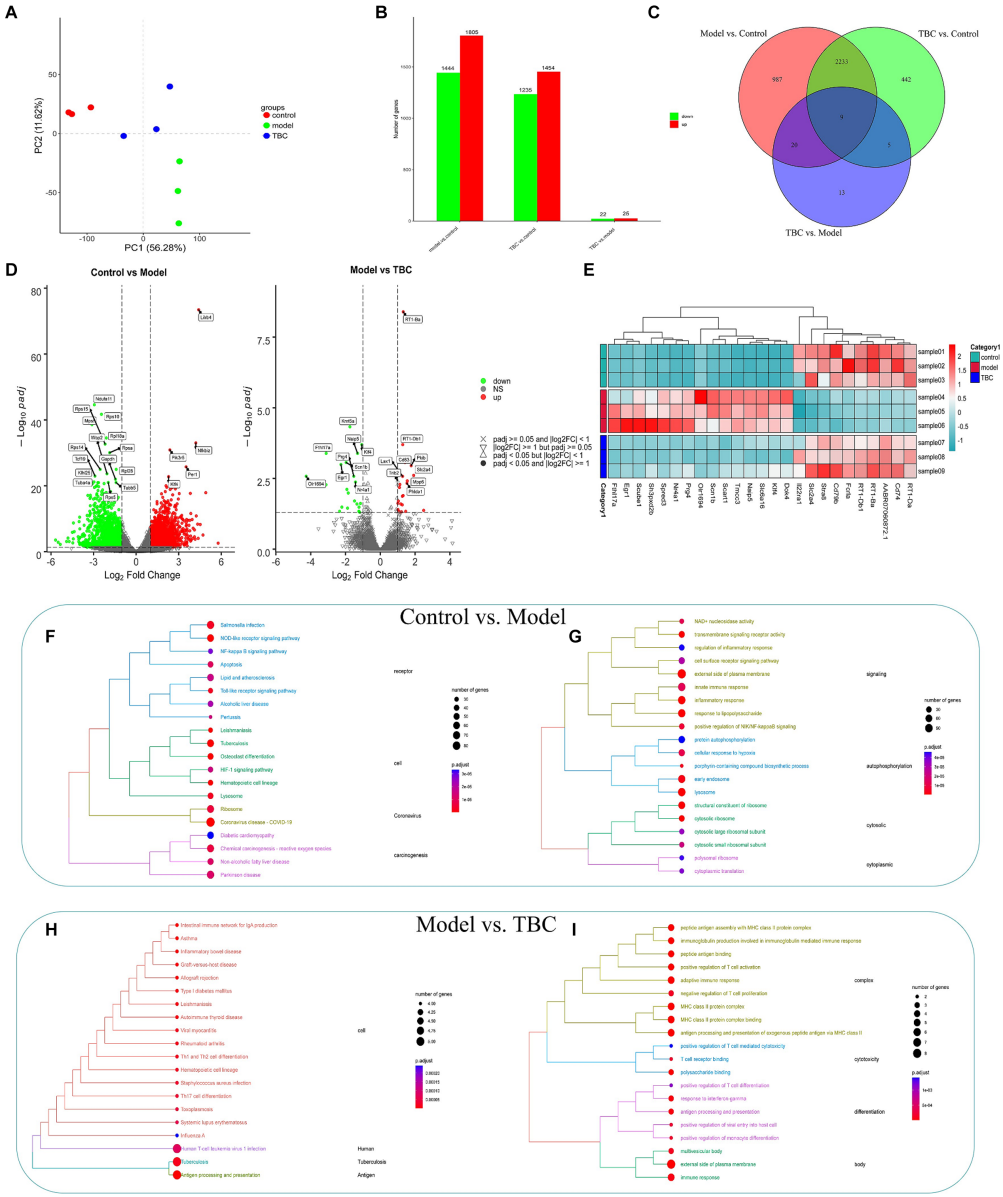
Effect of TBC on whole blood transcriptomics in diabetic LEAD rats. **(A)** Results of principal component analysis. **(B,C)** Number of TBC-regulated genes and number of intersections. **(D)** Differential gene volcano map. **(E)** Differential gene heat map. **(F,G)** KEGG and GO enrichment analysis of DEG in Model and Control groups (*p* < 0.05). **(F)** KEGG analysis. **(G)** GO enrichment analysis. **(H,I)** KEGG and GO enrichment analysis of DEG in TBC and model groups (*p* < 0.05). **(H)** KEGG analysis. **(I)** GO enrichment analysis.

To explore the potential mechanism of TBC treatment in diabetic LEAD rats, a transcriptomic analysis of whole blood was conducted. The TBC-H group was selected as a representative of the TBC intervention group for transcriptomic analysis. PCA score plot showed clear separation between the three groups, with the TBC group showing a trend to recovery compared with the control group, suggesting that there was a significant alteration in the whole blood transcriptome of the TBC-intervened diabetic LEAD rats (Figure 8A). The genes with a *Q* value ≤0.05 and |fold change| >1 were considered differentially expressed genes. The results showed that a total of 3,249 DEGs were identified in the model group compared with the control group, of which 1,805 genes were up-regulated and 1,444 genes were down-regulated. Forty-seven DEGs were detected in the TBC group compared with the model group, of which 25 were up-regulated and 22 were down-regulated (Figure 8B). The Venn diagram displayed 2,267 overlapping genes, among which 29 significantly up-or down-regulated genes in the model group were significantly restored by the TBC intervention (Figure 8C). The volcano plot captured the overall trend change in the number of DEGs in each group (Figure 8D). To clearly observe the changes in the differential genes in each group, the heatmap of the top 25 DEGs with the lowest *Q* values reversed by TBC treatment was drawn (Figure 8E). The DEGs were considered potential therapeutic targets of TBC in this study. Further KEGG pathway enrichment analysis and Gene Ontology (GO) analysis were performed on all DEGs. Compared with the control group, the model group mainly regulate the signaling pathways associated with human infectious diseases (coronavirus disease - COVID-19, tuberculosis, leishmaniasis, salmonella infection), immune regulation and inflammatory responses (NOD-like receptor signaling pathway, Toll-like receptor signaling pathway, HIF-1 signaling pathway, NF-kappa B signaling pathway), cell activation and synthesis processes (osteoclast differentiation, hematopoietic cell lineage), apoptosis, lipid and atherosclerosis-related (Figure 8F), involving various biosynthetic processes such as angiogenesis, immune-inflammatory response and cell growth (Figure 8G). Compared with the model group, the TBC intervention group was primarily involved in immunomodulation (antigen processing and presentation, intestinal immune network for IgA production), immune cell differentiation (Th1 and Th2 cell differentiation, Th17 cell differentiation, hematopoietic cell lineage), type I diabetes mellitus, and immune-related diseases (human T-cell leukemia virus 1 infection, systemic lupus erythematosus, rheumatoid arthritis, and autoimmune thyroid disease) (Figure 8H). Additionally, GO analysis showed that immune regulation, inflammatory response, and cell differentiation were significantly enriched in biological processes after TBC intervention, suggesting that TBC mainly treats diabetic LEAD rats by attenuating inflammatory response, promoting angiogenesis, and modulating immune response (Figure 8I).

### 3.6 Multi-omics correlation heatmap analysis

**Figure 9:**
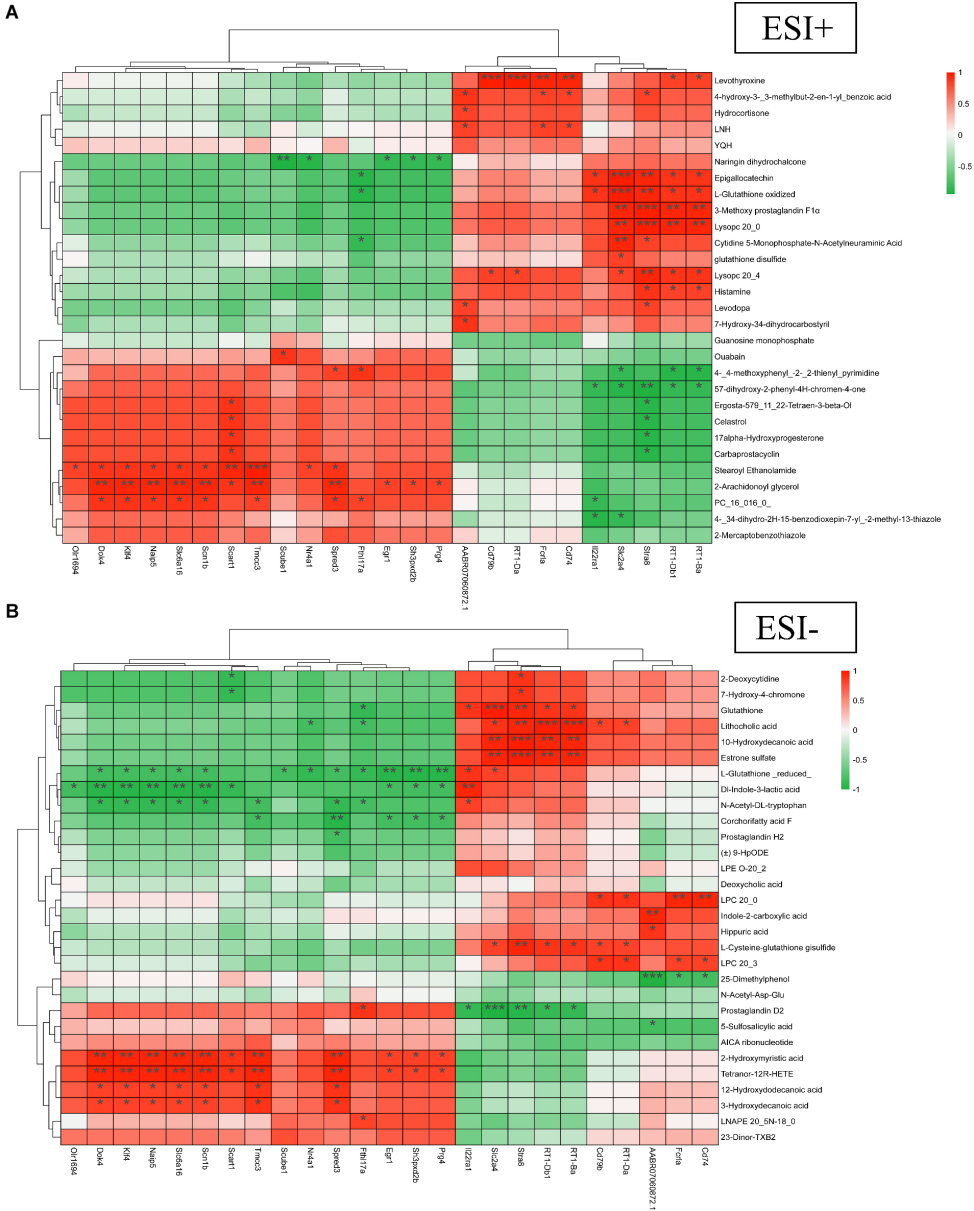
Multi-group correlation heatmap analysis of TBC on diabetic LEAD rats. **(A)** Positive ion. **(B)** Negative ion. Red for high positive correlation, green for high negative correlation. ^*^p < 0.05, ^**^p < 0.01, and ^***^p < 0.001.

Correlation heatmap analysis was performed based on relevant differential metabolites in the positive-negative ion model of metabolomics and the top 25 DEGs of potential reversal value in transcriptomics of diabetic LEAD rats with TBC intervention. The results showed that among the metabolites in positive ion mode, 2-Arachidonoyl glycerol showed a strong positive correlation with genes such as Dok4, Klf4, Naip5, Slc6a16, Scn1b, Tmcc3, and Spred3 (*p* < 0.01). Levothyroxine showed a strong positive correlation with Cd79b, RT1-Da, Fcrla, and Cd74 (*p* < 0.01). Epigallocatechin and L-Glutathione oxidized showed a strong positive correlation with Slc2a4 and Stra8 (*p* < 0.01), and 3-Methoxy prostaglandin F1α, Lysopc 20:0 showed a strong positive correlation with genes such as Slc2a4, Stra8, RT1-Db1 and RT1-Ba (*p* < 0.01). Naringin dihydrochalcone was in a strong negative correlation with genes such as Scube1 (*p* < 0.01). 5,7-dihydroxy-2-phenyl-4H-chromen-4-one was in strong negative correlation with Stra8 (*p* < 0.01) (Figure 9A).

In the negative ion mode, 10-Hydroxydecanoic acid and Estrone sulfate showed a strong positive correlation with genes such as Slc2a4, Stra8, RT1-Db1, and RT1-Ba (*p* < 0.01). Lithocholic acid showed strong positive correlation with Stra8, RT1-Db1, RT1-Ba, and other genes (*p* < 0.01). Glutathione showed strong positive correlation with Slc2a4 and Stra8 (*p* < 0.01). LPC 20:0 showed strong positive correlation with Fcrla and Cd74 (*p* < 0.01). 2-Hydroxymyristic acid and Tetranor-12R-HETE showed strong positive correlation with genes such as Dok4, Klf4, Naip5, Slc6a16, Scn1b, Tmcc3, and Spred3 (*p* < 0.01). L-Glutathione (reduced) showed a strong negative correlation with genes such as Egr1, Sh3pxd2b, and Prg4 (*p* < 0.01). Dl-Indole-3-lactic acid showed a strong negative correlation with genes such as Dok4, Klf4, Naip5, Slc6a16, and Scn1b (*p* < 0.01). Prostaglandin D2 showed strong negative correlation with genes such as Slc2a4, and Stra8 (*p* < 0.01) (Figure 9B). The correlation analysis revealed a close relationship between differential genes and metabolites.

## 4 Discussion

**Figure 10:**
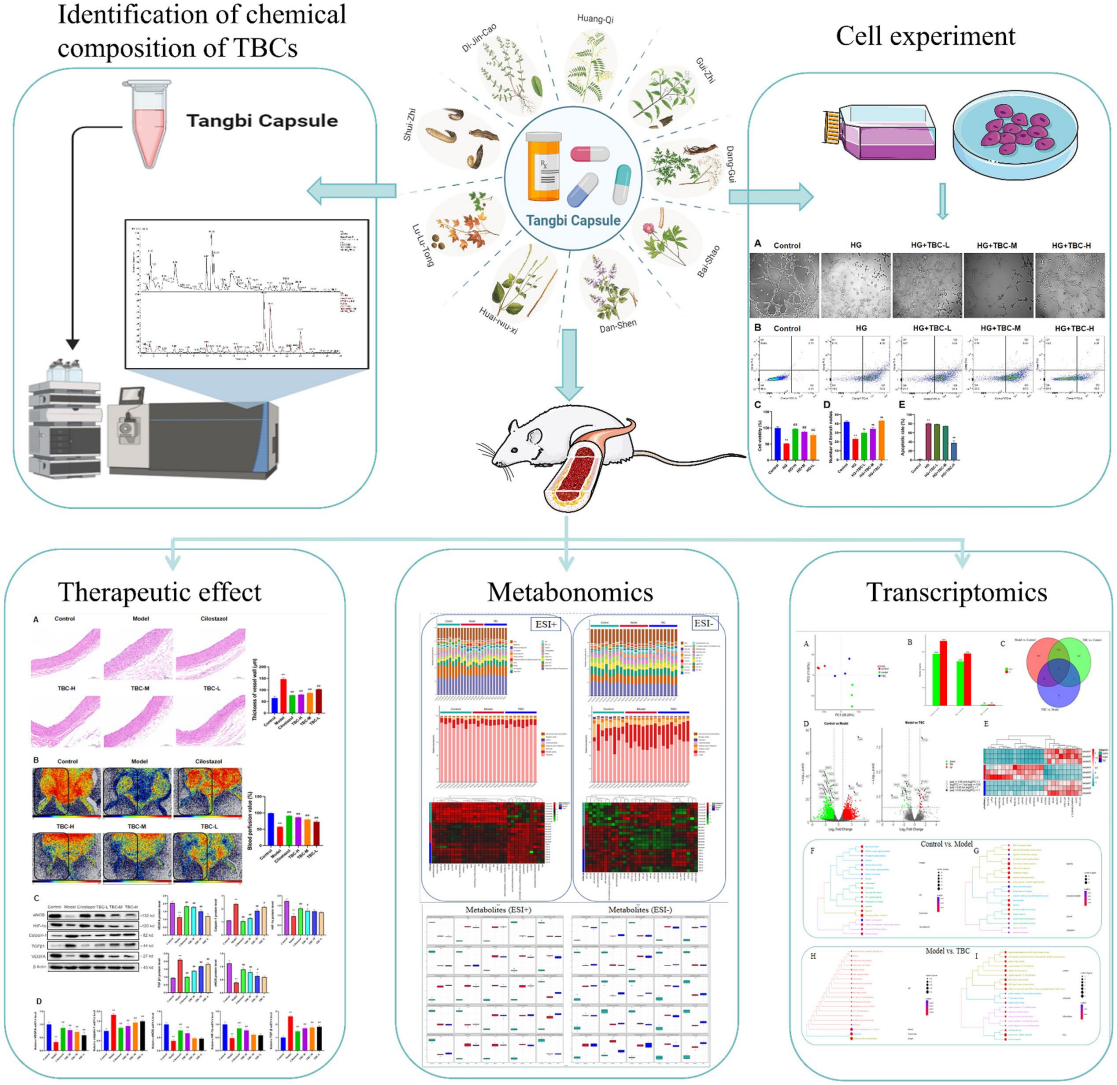
The flowchart of this study of Tangbi capsule on diabetic lower extremity vascular disease.

Diabetic LEAD is a serious complication of diabetes mellitus patients, which seriously affects their life and health. The development of Diabetic LEAD is closely related to oxidative stress, dyslipidemia, atherosclerosis, chronic inflammatory injury, and vascular endothelial dysfunction (Brevetti et al., 2008; Nativel et al., 2018b). Increasing clinical and experimental evidence suggests that TCM plays an important role in the treatment of diabetes and diabetic LEAD. Nevertheless, the involvement of traditional Chinese medicine in diabetic LEAD mostly concentrates on investigating monomeric substances and falls short of offering a thorough assessment of treatment outcomes. Furthermore, angiogenesis is frequently used to examine the potential therapeutic benefits of traditional Chinese medicine formulae like Danshen Injection for diabetic LEAD (Li et al., 2004; He et al., 2016). The synergistic effect of multiple traditional Chinese medicine ingredients may provide a comprehensive therapeutic effect and lessen the limitations of single drug treatment. TBC can treat diabetic LEAD by improving blood circulation, anti-inflammatory response, and antioxidant mechanisms. This study investigated the efficacy, chemical composition, mechanism, and metabolic regulation of the TCM formula TBC in diabetic LEAD rats (Figure 10). The results showed that TBC improved vascular endothelial injury, apoptosis, lipid accumulation, liver and kidney functions, and restored lower extremity blood flow in diabetic LEAD rats. In addition, the mechanism of TBC in the treatment of diabetic LEAD may be related to the modulation of inflammatory immunity, lipid metabolism, and amino acid metabolism. The active chemical components of TBC include (2R)-2,3-Dihydroxypropanoic acid, catechin, citric acid, miquelianin, carminic acid, salicylic acid, and formononetin.

Results demonstrated that TBC improved vascular endothelial damage and restored blood flow to the lower extremities in rats with diabetic LEAD. Histologic and biochemical experiments on the femoral artery confirmed that TBC improved vascular injury, lipid accumulation, and liver and kidney function. In addition, TBC reduced the expression of inflammatory factors (IL-6, TNF-α, IL-1β) and attenuated the inflammatory response. Chronic inflammatory damage promotes the formation of glycosylation products, which in turn causes abnormal protein cross-linking, leading to vascular wall thickening, decreased vascular elasticity, and vascular lumen narrowing. IL-6 plays an important role in regulating the inflammatory response that causes damage to the vascular wall and accelerates atherosclerosis (Zegeye et al., 2018). TNF-α is an important pro-inflammatory factor, and previous studies have shown that TNF-α can impair endothelial function, induce atherosclerosis and insulin resistance, accelerate the proliferation of smooth muscle cells, and promote the release of platelet growth factor from platelets, accelerating the progression of vascular lesions (Buckley et al., 2005). IL-1β is a pleiotropic cytokine that increases collagen production in a dose-dependent manner and modulates innate immunity by inducing the release of other inflammatory mediators (Wang et al., 2011). Thus, TBC can reduce the inflammatory response by decreasing the expression of inflammatory factors such as IL-6, TNF-α, and IL-1β, which in turn improves vascular injury and stenosis and restores the blood flow in the lower extremities.

Results also showed that TBC significantly increased the levels of protein and expression of angiogenesis-related factors including HIF-1α, eNOS, and VEGFA. However, the protein and gene expression of Calpain-1 and TGFβ were significantly inhibited, indicating that TBC promoted angiogenesis, regulated reactive oxygen species, and attenuated endothelial damage. Results of *in vitro* cellular experiments showed that TBC intervention enhanced endothelial cell activity, reduced apoptosis, and enhanced endothelial cell angiogenesis. Angiogenesis refers to the formation of new blood vessels from pre-existing vascular systems and it is controlled by various angiogenic and anti-angiogenic factors in addition to multiple signaling pathways (Dudley and Griffioen, 2023). VEGF is a potent angiogenic factor, and VEGFR2 on the surface of endothelial cells triggers endothelial cell proliferation, survival, and migration to induce angiogenesis (Lopes-Coelho et al., 2021). One of the most important functions of atherogenesis is to increase arterial diameter by upregulating smooth muscle cell proliferation through MCP-1 recruitment of immune cells and triggering inflammation. Immune cells recruited to sites of inflammation are a source of atherogenic factors (e.g., TGFβ and HIF-1α) that promote vascular smooth muscle cell proliferation to form new arteries and restore limb perfusion (Niu et al., 2008). Studies have shown that administration of an adenovirus encoding a constitutively active form of HIF-1α increases blood flow recovery in aged mice to levels similar to young mice (Bosch-Marce et al., 2007). In addition, another important factor in collateral formation is eNOS, whose expression is significantly increased in collateral vessels during arteriogenesis. eNOS-mediated NO release plays an important role in maintaining vascular homeostasis (Zhang et al., 2022). Clinical studies demonstrated that impaired NO-induced vasodilatation mediated by eNOS led to structural and functional changes in subcutaneous arterioles and thickened vessel walls (Hsu et al., 2016). In conclusion, in rats with diabetic LEAD, TBC intervention can promote angiogenesis by regulating the expression of angiogenesis-related factors such as HIF-1α, eNOS, and VEGFA.

Metabolomic analysis elucidated the complex pathophysiological processes involved in the treatment of diabetic LEAD with TBC and 87 different metabolites were identified in this study through serum untargeted metabolomic analysis. Pathway enrichment analysis showed that these metabolites may be involved in the treatment of diabetic LEAD by regulating core metabolic pathways such as arginine biosynthesis, phenylalanine metabolism, pyrimidine metabolism, arachidonic acid metabolism, nucleotide metabolism, and vitamin B6 metabolism. Arginine biosynthesis can be converted into NO and indirectly dilate blood vessels and promote the restoration of blood circulation (Morris, 2016). Phenylalanine metabolism can promote glycolipid metabolism and reduce lipid accumulation (Shrode et al., 2022). Pyrimidine is an important biomolecule involved in cellular energy metabolism and DNA synthesis. Regulation of pyrimidine metabolism improves endothelial cell damage and death. Arachidonic acid metabolism regulates cholesterol levels, reduces thrombosis, and lowers lipid levels (Ward, 1991). Vitamin B6 metabolism can protect vascular endothelial cells, reduce endothelial cell activation and platelet damage, inhibit platelet agglutination, blood coagulation, platelet production of prostaglandins, and promote the production of cyclic prostaglandins in vascular endothelial cells (Austin et al., 2004). The results of this study showed that TBC improved dyslipidemia and vascular injury in rats with diabetic LEAD. Transcriptomic analysis further explored the effect of TBC on diabetic LEAD, revealing that TBC could affect immune-related functional pathways. GO results suggested that TBC could promote angiogenesis and reduce inflammatory response. In addition, correlation analysis showed that the differential key genes and key metabolites were significantly associated with glucose-lipid metabolism and amino acid metabolic processes, indicating that TBC treatment improved the metabolic processes of the diabetic LEAD rats.

With liquid mass spectrometry, this study clarified the active chemical components of TBC to investigate its mechanism of action. Identified components include (2R)-2,3-Dihydroxypropanoic acid, catechin, citric acid, miquelianin, carminic acid, salicylic acid, and formononetin. Among them, (2R)-2,3-Dihydroxypropanoic acid can increase arterial blood flow and improve blood supply by acting on vascular smooth muscle and causing vasodilatation, particularly in arteries (Lourenco et al., 2009). The phenolic hydroxyl group of catechin can terminate the reaction with reactive oxygen and nitrogen species, breaking the cycle of generation of new free radicals to stop the free radical chain reaction and prevent the oxidation of cellular lipids (Fraga et al., 2010), and reducing the accumulation of cholesterol and its oxidized products in the arterial wall to improve blood circulation (Suzuki et al., 2005). Catechin also alleviates conditions associated with vascular dysfunction, including vascular inflammation, smooth muscle cell proliferation, platelet aggregation, lipoprotein oxidation, lipid alterations, and vascular reactivity (Velayutham et al., 2008). In addition, catechin reduces the expression of cytokines of inflammation, NF-κB, intercellular adhesion molecule 1, and TNF-α, thereby improving the inflammatory response (Suzuki et al., 2007). Citric acid is thought to have anticoagulant properties due to its ability to chelate Ca^2+^ ions (Oudemans-van Straaten and Ostermann, 2012). Citric acid can reduce endothelial dysfunction and inflammation and promote endothelial cell sprouting and angiogenic factor production (Binu et al., 2013). Miquelianin has antidiabetic potential such as antioxidant, antiglycation, and aldose reductase inhibition (Zuo et al., 2021). Carminic acid possesses a variety of bioactivities that protect erythrocytes and DNA from free radical-induced oxidation (Li et al., 2009). Carminic acid can scavenge free radicals by mediating the TNF-α/TNFRs and Nrf-2 signaling pathways to inhibit inflammation and oxidative stress and reduce lipid deposition (Li et al., 2022). Salicylic acid is one of the most commonly used anti-inflammatory reagents and is the major *in vivo* metabolite of aspirin, known to inhibit the inflammatory response and protect endothelial cells by preventing endothelial mitochondrial fragmentation (Forrester et al., 2020). Formononetin is a bioactive isoflavone with antioxidant and anti-inflammatory properties (Aladaileh et al., 2019). Interestingly, formononetin can improve arterial endothelial function by up-regulating eNOS via the estrogen receptor and mitogen-activated protein kinase pathways and may play an active role in reducing thrombosis in the lower extremities (Sun et al., 2016).

The study investigated the efficacy and mechanism of TBC on diabetic LEAD through *ex vivo* experiments, chemical composition analysis, metabolomics, and transcriptomics, offering preliminary evidence to guide the use of TBC as a therapy option for diabetic LEAD. However, certain limitations should be considered. Firstly, this study was qualitative and quantitative test was not performed to analyze the specific components. Secondly, in-depth validation results of transcriptomics was not applied and the effects of differential genes on diabetic LEAD rats should be further investigated.

## 5 Conclusion

TBC improved vascular endothelial injury, apoptosis, lipid accumulation, liver and kidney function, and restored blood flow in the lower extremities of diabetic LEAD rats. The mechanism of TBC in the treatment of diabetic LEAD may be related to the modulation of inflammatory immunity, lipid metabolism, and amino acid metabolism. The active ingredients of TBC include (2R)-2,3-Dihydroxypropanoic acid, catechin, citric acid, miquelianin, carminic acid, salicylic acid, and formononetin. This study offers preliminary evidence to guide the application of TBC as a therapy for diabetic LEAD. However, quantified detection of specific drug components was not performed and the transcriptomics results were not fully validated. Given the limitations, the results should be established or confirmed on a larger scale with more precise instructions in future investigations.

## Data availability statement

The datasets presented in this study can be found in online repositories. The names of the repository/repositories and accession number(s) can be found in the article/Supplementary material.

## Ethics statement

The animal study was approved by Experimental Animal Ethics Committee of Guangdong Hospital of Chinese Medicine. The study was conducted in accordance with the local legislation and institutional requirements.

## Author contributions

Y-hL: Conceptualization, Data curation, Formal analysis, Methodology, Writing – original draft. D-wW: Formal analysis, Visualization, Writing – original draft. JY: Resources, Visualization, Writing – original draft. YH: Formal analysis, Writing – original draft. LZ: Formal analysis, Investigation, Resources, Writing – original draft. J-xW: Investigation, Writing – review & editing. J-mZ: Investigation, Writing – review & editing. L-LW: Resources, Writing – original draft. G-jF: Methodology, Project administration, Resources, Writing – review & editing. WS: Conceptualization, Formal analysis, Funding acquisition, Methodology, Project administration, Resources, Supervision, Writing – review & editing.

## Footnotes

^1^ https://www.bioincloud.tech/

^2^ https://hmdb.ca/metabolites

^3^ http://www.lipidmaps.org/

^4^ https://www.genome.jp/kegg/pathway.html
