# Supplementary material for: Integrated metabolomics and transcriptomics analysis reveals the mechanism of Tangbi capsule for diabetic lower extremities arterial disease
Source: Front Microbiol. 2024 Jul 22;15:1423428. doi: 10.3389/fmicb.2024.1423428 (PMC11299497; doi:10.3389/fmicb.2024.1423428)
Supplement: Supplementary file 1 [file Table_1.DOCX]

Supplementary Material

## Figure 1 Total ion current map for identification of natural products in TBC samples

Note: Black in column 1 is the total ion flow diagram of the negative ion mode, and red in column 2 is the total ion flow diagram of the positive ion mode

**Table 1: Determination of active chemical components of TBC based on liquid chromatography**

| NO | Rt（min） | Identification | Formula | MW | m/z |
| --- | --- | --- | --- | --- | --- |
| 1 | 1.34 | Succinic semialdehyde | C4 H6 O3 | 102.03169 | [M-H]-119.03346 |
| 2 | 1.40 | D-(+)-Maltose | C12 H22 O11 | 342.11621 | [M-H+HAc]-387.11374 |
| 3 | 1.63 | (2R)-2,3-Dihydroxypropanoic acid | C3 H6 O4 | 106.02661 | [M-H]-105.01778 |
| 4 | 1.79 | D-(+)-Malic acid | C4 H6 O5 | 134.02152 | [M-H]-133.01282 |
| 5 | 2.63 | 4-Oxoproline | C5 H7 N O3 | 129.04259 | [M-H]-128.03392 |
| 6 | 5.19 | Pyrogallol | C6 H6 O3 | 126.03169 | [M-H]-125.02303 |
| 7 | 8.98 | Catechin | C15 H14 O6 | 290.07904 | [M-H]-289.07162 |
| 8 | 9.92 | Citric acid | C6 H8 O7 | 192.027 | [M-H]-191.01869 |
| 9 | 10.62 | [(3R,5R,6S,8S)-3-(β-D-Glucopyranosyloxy)-6-hydroxy-8-methyl-9,10-dioxatetracyclo[4.3.1.02,5 03,8]dec-2-yl]methyl benzoate | C23 H28 O11 | 480.16316 | [M+FA-H]-525.16028 |
| 10 | 10.99 | N'5-[3-(trifluoromethyl)benzoyl]-2,1,3-benzoxadiazole-5-carbohydrazide | C15 H9 F3 N4 O3 | 350.06267 | [M-H]-349.05933 |
| 11 | 11.63 | Suberic acid | C8 H14 O4 | 174.08921 | [M-H]-173.08087 |
| 12 | 12.47 | Miquelianin | C21 H18 O13 | 478.07474 | [M-H]-477.06723 |
| 13 | 12.54 | Carminic acid | C22 H20 O13 | 492.09039 | [M-H]-491.08298 |
| 14 | 12.87 | Salicylic acid | C7 H6 O3 | 138.03169 | [M-H]-137.02306 |
| 15 | 13.95 | Oleonuezhenide | C48 H64 O27 | 1072.3635 | [M+FA-H]-1117.36121 |
| 16 | 14.46 | Formononetin | C16 H12 O4 | 268.07356 | [M-H]-267.06601 |
| 17 | 15.78 | Corchorifatty acid F | C18 H32 O5 | 328.22497 | [M-H]-327.21738 |
| 18 | 16.33 | Dodecanedioic acid | C12 H22 O4 | 230.15181 | [M-H]-229.14398 |
| 19 | 17.78 | (+/-)9-HpODE | C18 H32 O4 | 312.23006 | [M-H]-311.22269 |
| 20 | 18.89 | Avermectin B1a | C48 H72 O14 | 872.49221 | [M-H]-871.46899 |
| 21 | 19.44 | 2-Hydroxymyristic acid | C14 H28 O3 | 244.20384 | [M-H]-243.19617 |
| 22 | 20.34 | Labdanolic acid | C20 H36 O3 | 324.26644 | [M-H]-323.25888 |
| 23 | 21.25 | Gallic acid | C7 H6 O5 | 170.02152 | [M-H]-169.01309 |
| 24 | 21.84 | Arachidonic acid | C20 H32 O2 | 304.24023 | [M-H]-303.23257 |
| 25 | 22.44 | Palmitic acid | C16 H32 O2 | 256.24023 | [M-H]-255.23257 |
| 26 | 22.67 | Oleic acid | C18 H34 O2 | 282.25588 | [M-H]-281.24847 |
| 27 | 23.40 | Docosatrienoic acid | C22 H38 O2 | 334.28718 | [M-H]-333.27975 |
| 28 | 23.70 | Stearic acid | C18 H36 O2 | 284.27153 | [M-H]-283.26398 |
| 29 | 24.04 | 11(Z)-Eicosenoic acid | C20 H38 O2 | 310.28718 | [M-H]-309.27982 |
| 30 | 25.59 | Erucic acid | C22 H42 O2 | 338.31848 | [M-H]-337.31113 |
| 31 | 25.67 | Oleanolic acid | C30 H48 O3 | 456.36035 | [M-H]-455.35303 |
